# Extracellular Vesicle-Mediated Modulation of Stem-like Phenotype in Breast Cancer Cells under Fluid Shear Stress

**DOI:** 10.3390/biom14070757

**Published:** 2024-06-25

**Authors:** Spenser R. Brown, Margaret E. Radcliffe, Joseph T. Danner, Wilmer J. Andújar Cruz, Kimberly H. Lackey, Han-A Park, Steven T. Weinman, Yonghyun Kim

**Affiliations:** 1 Department of Chemical and Biological Engineering, The University of Alabama, Tuscaloosa, AL 35487, USAstweinman@eng.ua.edu (S.T.W.); 2 Department of Biological Sciences, The University of Alabama, Tuscaloosa, AL 35487, USA; lacke003@ua.edu; 3 Department of Human Nutrition and Hospitality Management, The University of Alabama, Tuscaloosa, AL 35487, USA; hpark36@ches.ua.edu

**Keywords:** extracellular vesicles, breast cancer metastasis, cancer stem cells, fluid shear stress

## Abstract

Circulating tumor cells (CTCs) are some of the key culprits that cause cancer metastasis and metastasis-related deaths. These cells exist in a dynamic microenvironment where they experience fluid shear stress (FSS), and the CTCs that survive FSS are considered to be highly metastatic and stem cell-like. Biophysical stresses such as FSS are also known to cause the production of extracellular vesicles (EVs) that can facilitate cell–cell communication by carrying biomolecular cargos such as microRNAs. Here, we hypothesized that physiological FSS will impact the yield of EV production, and that these EVs will have biomolecules that transform the recipient cells. The EVs were isolated using direct flow filtration with and without FSS from the MDA-MB-231 cancer cell line, and the expression of key stemness-related genes and microRNAs was characterized. There was a significantly increased yield of EVs under FSS. These EVs also contained significantly increased levels of miR-21, which was previously implicated to promote metastatic progression and chemotherapeutic resistance. When these EVs from FSS were introduced to MCF-7 cancer cells, the recipient cells had a significant increase in their stem-like gene expression and CD44^+^/CD24^−^ cancer stem cell-like subpopulation. There was also a correlated increased proliferation along with an increased ATP production. Together, these findings indicate that the presence of physiological FSS can directly influence the EVs’ production and their contents, and that the EV-mediated transfer of miR-21 can have an important role in FSS-existing contexts, such as in cancer metastasis.

## 1. Introduction

Metastasis is the leading cause of cancer deaths worldwide [1], with metastatic breast cancer being the second deadliest cancer among women, behind lung cancer [2]. Metastatic breast cancer presents additional treatment challenges since the cells at the secondary site have evolved from the phenotype of the primary tumor. These cells have traveled as circulating tumor cells (CTCs), which affects their ability to adapt to the dynamic environment during circulation and in secondary site colonization. While in circulation, CTCs can encounter fluid shear stress (FSS) and immunological responses [3]. Although most CTCs die in circulation, some of them survive with the potential to form distant metastases [4] and are thus targeted for diagnostics [5,6]. We and others theorized that this subset of CTCs overlaps with the cancer stem cell (CSC) subpopulation [7,8,9,10], which can help explain how these cells are adaptable to dynamic environments. CSCs are known to be capable of self-renewal, differentiation, tumor initiation, invasion, and metastatic cancer propagation [11]. They are known to possess chemotherapeutic resistance through efflux pumps that remove would-be toxins from the cytosol and via extracellular vesicles (EVs) [12,13]. CSCs are also plastic, being able to convert from stem cell state to a non-stem state when the need arises [14]. It is lesser known how such plasticity is conferred and passed to other differentiated cells, especially in stressful dynamic environments. Since stem-like behavior enables metastasis, it is of significant research interest to better understand the mechanisms of perpetuating stemness qualities.

EVs facilitate cell-to-cell communication through the transfer of biomolecules. EVs are lipid bilayer vesicles that contain membrane-penetrating proteins, receptors, and nucleic acids [13]. They are formed by cells as a result of inward budding during endosomal maturation [15]. There are several subsets of EVs including exosomes, endosomes, and apoptotic bodies. Exosomes are 50–150 nm in diameter and are capable of influencing angiogenesis, facilitating movement through tissues, and promoting cancer cell growth [16,17]. Further, EVs have demonstrated the abilities to confer cancer-like qualities on normal cells such as fibroblasts and epithelial cells [18] and carry the integrins that direct organ-specific metastases [19]. These abilities are attributed to the transfer of genetic cargo from the EVs to recipient cells. EVs are also thought to nurture the “soil” in the “seed-and-soil” hypothesis [20] for metastatic spread [21,22]. Various stresses including hypoxia, serum starvation, and thermal stress applied to EV-producing cells have induced aberrations in the amounts of protein and DNA expressed within the EVs produced under stress [23,24,25,26]. Others have acknowledged that EV-mediated preconditioning could be a key factor in tumor progression by conveying stress-induced responses [27].

It has been demonstrated that EVs (namely exosomes) derived from stem cells have a variant microRNA (miRNA) profile that is distinct from that of EVs isolated from non-stem cells [28]. Additionally, miRNAs that are found in EVs can enhance tumor invasiveness and metastasis [29]. Notably, miR-21 and miR-29a were identified as promoting breast CSC phenotypes and being potential biomarkers of breast and lung cancer cells [29,30,31,32]. Though it has been shown that the application of FSS has induced the rapid production of microparticles [33], to the best of our knowledge, the effects of FSS on the composition of EVs has not been extensively reported. We hypothesize that (1) FSS influences the composition of EVs and (2) EVs from CSC-like cells (i.e., cells with a high percentage of CD44^+^/CD24^−^ subpopulation) will endow a stem-like phenotype on the recipient cells. Illuminating the relationship between EVs and the commutation of stem-like traits will provide insight for developing targeted therapeutics. To elucidate the impact that FSS has on stem-like phenotype via EVs, we focus on miRNAs that correlate to stemness: miR-21 induces targeted therapy resistance, mediates metastasis, and is upregulated in stem cell-derived EVs [13,16,28]; miR-16 is involved in endocrine and targeted therapy resistances [13]; miR-17 has been implicated in mediating metastasis [13,16]; and miR-29a is correlated with tumorigenicity, drug resistance, and metastasis, promoting proliferation, and has been shown to be upregulated in stem cell-derived EVs [28,32]. Here, we present evidence that FSS directly impacts the production and composition of EVs, which subsequently increases the stem-like phenotype and proliferation of recipient cells.

## 2. Materials & Methods

### 2.1. Cell Culture

MDA-MB-231 and MCF-7 cells were obtained from ATCC (Manassas, VA, USA). MCF-7 was cultured in phenol red-free Dulbecco’s modified Eagle’s medium (DMEM) (Gibco, Grand Island, NY, USA) supplemented with 10% fetal bovine serum (Gibco), 1% penicillin/streptomycin (Corning, Corning, NY, USA), and 1% amphotericin B (Cytiva, Marlborough, MA, USA). For EV incubation studies, the FBS was pre-depleted of native EVs by ultracentrifugation (100,000× *g* for 14 h). MDA-MB-231 was cultured in DMEM/F12 (Gibco) supplemented with 1X B-27 (Gibco), 1% penicillin/streptomycin (Corning), 5 mg/L insulin (Thermo Scientific, Waltham, MA, USA), 20 µg/L bFGF (Shenandoah Inc., Warwick, PA, USA), 20 µg/L EGF (Shenandoah Inc.), 0.5 mg/L hydrocortisone (Sigma-Aldrich, St. Louis, MO, USA), 8 µg/mL heparin (Akron Biotech, Boca Raton, FL, USA), and 1% amphotericin B (Cytiva). Cell viability was measured using trypan blue staining (Amresco, Solon, OH, USA) and counted using a T20 automated cell counter (Bio-Rad, Hercules, CA, USA).

### 2.2. Fluid Shear Stress

Cells were inoculated into a CLS-1450 Series spinner flask (Chemglass Life Sciences, Vineland, NJ, USA). The flasks were incubated on a Dura-Mag magnetic stirrer (Chemglass Life Sciences) for 4 h at a wall FSS of 5 dyn/cm^2^. FSS was calculated using the correlation equations of power number for the propeller [34]. For each FSS, 6 × 10^6^ cells were inoculated in 60 mL of media and cultured in T-75 flasks for 24 h post-FSS to allow cell recovery prior to media collection for EV isolation.

### 2.3. EV Isolation

EVs collected via ultracentrifugation followed a differential centrifugation protocol. Conditioned cell medium was centrifuged using a high-speed centrifuge (Avanti J-30I; Beckman Coulter, Brea, CA, USA). The conditioned media were centrifuged at 300× *g* for 5 min, 1000× *g* for 5 min, 4000× *g* for 10 min, 20,000× *g* for 20 min, and, finally, at 100,000× *g* for 60 min. The supernatant was transferred to a new tube following each spin, until, ultimately, the pelleted EVs were collected after the final step.

EVs were collected by direct flow filtration (DFF), where conditioned cell culture medium was centrifuged at 1000× *g* for 10 min and, subsequently, at 10,000× *g* for 10 min to remove remaining cell debris, extracellular proteins, and apoptotic bodies. Synder LY (PES, 100 kDa, Synder Filtration, Vacaville, CA, USA) membranes were soaked in DI H_2_O in a 4 °C refrigerator overnight. Each membrane coupon (active area = 14.6 cm^2^) was cut and loaded into an HP4750 direct flow filtration cell (Sterlitech, Auburn, WA, USA). The filtration cell was filled with 50 mL of DI H_2_O and pressurized to 6.9 bar_g_ for 1 min to facilitate pore wetting. Afterwards, excess water was removed from the filtration cell and replaced with 20 mL of conditioned cell media. A complete filtration (i.e., no stirring) of the cell media was performed at a pressure of 2 bar_g_ for approximately 2.5 h. Remaining cell media (<1 mL) were removed with a pipettor prior to disassembling the filtration cell. The membrane coupon was removed from the filtration cell and placed in a sterilized petri dish where the EVs were eluted from the membrane in 5 mL of 1X PBS following a 45-min incubation at 4 °C. Lastly, the collected EVs were filtered using a 200 nm Avanti extruder five times.

### 2.4. Scanning Electron Microscopy (SEM)

Membrane top surface morphology was imaged with an Apreo S field-emission scanning electron microscope (FE-SEM, Thermo Fisher Scientific, Waltham, MA, USA). Pristine samples were dehydrated using ethanol solutions consisting of 25, 50, 75, and ~100% ethanol for 5 min apiece in a stepwise manner. Each membrane was stored in ~100% ethanol until the sample was imaged. Prior to mounting, the dehydrated membrane was transferred to a solution containing 50% HMDS/50% ethanol (*v*/*v*) for 15 min, and then it was soaked in a 100% HMDS solution for at least 15 min to prevent pore collapse [35]. Next, the membranes were dried in air for 5 min and mounted to an aluminum stub mount with carbon tape, followed by placing a piece of conductive copper tape across the sample and stub mount to form a direct connection. Samples were sputter coated with an MCM-200 Ion Sputter Coater (SEC, Hwaseong-si, Republic of Korea) with a ~5 nm layer of gold before they were placed inside the FE-SEM. Images were acquired using the Optiplan lens and T1 segment A detector at a working distance of 3–5 mm, a magnification of 65,000×, an accelerating voltage of 5.00 kV, a beam current of 25 pA, and a scan speed of 5.00–10.00 µs.

Top surface FE-SEM images were analyzed using Fiji image processing package (version 2.14.0) [36] and ImageJ image processing software (version 1.54f) [37] to estimate the pore size distribution of the membrane [38,39]. Similar to our previous work [40], each image was converted to an 8-bit format and the software was calibrated using the micrograph scale bar before further processing. The color threshold was adjusted to provide adequate contrast for the analyze particles function. The analyze particles function was used to calculate Feret’s diameter for top surface pores between a range of 10 and 1000 nm. Feret’s diameter provides the approximate circular pore diameter based on the longest distance between any two points of each pore [41]. Results were exported to OriginPro 2023 (version 10.0.0.154) to generate the pore size distribution.

### 2.5. EV Verification and Quantification

The presence of EVs was qualitatively verified by transmission electron microscopy (TEM) on a FEI TECNAI F20 TEM. Samples were fixed in 4% paraformaldehyde in PBS (Fisher Scientific, Hampton, NH, USA) for 30 min and loaded onto a carbon coated copper grid. Samples were allowed to dry for at least 20 min, stained with 2% uranyl acetate for 5 min, and washed with ultra-filtered DI H_2_O.

The presence of EVs was verified by protein analysis using the commercial Exo-Check Exosome Array kit (System Biosciences, Palo Alto, CA, USA) according to the manufacturer’s protocol. For imaging, the membrane was treated with the SuperSignal West Pico PLUS Chemiluminescent Substrate system (Thermo Scientific) for imaging on the ChemiDoc MP Imaging System with ImageLab software (v.5.1).

For total protein quantifications, EVs isolated with methods described above were resuspended 9:1 (*v*/*v*) in 100% (200 proof) ethanol at −80 °C for a minimum of 2 h to precipitate the proteins. The precipitated protein was centrifuged at 3220× *g* for 1 h to concentrate the precipitate. The ethanolic supernatant was aspirated and the pellet allowed to air dry before being resuspended in RIPA lysis buffer. BSA standards were used for calibration and all protein samples were quantified via BCA assay measured at 562 nm. EV protein collected was normalized to the cell number that produced the conditioned medium.

### 2.6. Nanoparticle Tracking Analysis (NTA)

To determine the size and quantity of the EVs, NTA was performed with a NanoSight NS-300 instrument running the NTA 3.4 software (Malvern Panalytical, Malvern, UK). Samples were kept in PBS on ice until NTA was performed. In volumes of 1 mL, samples were loaded in the sample chamber and the camera was focused. For all recordings, a cameral level of 9 and screen gain of 9 was used. Five 60 s videos were recorded for each sample and each sample was loaded at a flow rate of 50. Averages of particle size and quantity from the recordings were calculated (*n* = 5).

### 2.7. qRT-PCR

EV miRNAs were isolated via Qiagen’s (Hilden, Germany) miRNeasy Serum/Plasma kit according to the manufacturer’s protocol. Briefly, 1 mL of QIAzol Lysis Reagent was added to 200 µL suspended EVs in 1X PBS and vortexed to mix. After a 5-min incubation, 200 µL chloroform was added to the mixture and shaken thoroughly for 15 s. After a 3 min incubation, the lysate was centrifuged at 12,000× *g* for 15 min at 4 °C. The upper aqueous solution was transferred to a new collection tube and supplemented with 1.5 volumes of 100% ethanol. The sample was loaded into a RNeasy MinElute spin column and centrifuged at 10,000× *g* for 30 s until all of the lysate had passed through the column. The spin column was washed with two separate provided buffers followed by 80% ethanol. The column was centrifuged at maximum speed for 5 min to dry the membrane prior to eluting the miRNA in 14 µL RNase-free water. miRNA was quantified on a NanoDrop 2000. Complementary DNA (cDNA) was synthesized (35 ng/µL miRNA) using the Qiagen miRCURY LNA RT kit according to the manufacturer’s protocol. qRT-PCR was performed using the Qiagen miRCURY LNA miRNA SYBR Green PCR kit according to the manufacturer’s protocol. Primers for specific miRNA were acquired from Qiagen via the GeneGlobe database: miR-21 (GeneGlobe ID YP-00204230); miR-16 (GeneGlobe ID YP-00205702); miR-17 (GeneGlobe ID YP-02119304); miR-29a (GeneGlobe ID YP-00204698). Expressions of miRNA of interest were normalized to hsa-miR-30e-5p (GeneGlobe ID YP-204714) as a reference marker [42].

Cell-derived RNA was isolated with a GeneJet RNA Purification kit (Thermo Scientific) according to the manufacturer’s protocol for mammalian cultured cells. RNA was quantified on a NanoDrop 2000. cDNA was synthesized with the qScript cDNA SuperMix (Quanta Biosciences, Gaithersburg, MD, USA) and Mastercycler Nexus Gradient (Eppendorf, Hauppauge, NY, USA) according to the manufacturer’s protocol. Applied Biosystems PowerUp SYBR Green Master Mix was used in concordance with the manufacturer’s instructions for real-time PCR. A StepOnePlus Real-Time PCR System was used for all qRT-PCR and the StepOne Software (v2.3) was used for data analysis. Data were processed using the ΔCt method to measure the changes in gene and miRNA expression. The NCBI gene database was used to select the primer sequences as shown in Table 1.

### 2.8. Fluorescent Imaging

Isolated EVs were stained with PKH67 Green Fluorescent Cell Linker Kit for General Cell Membrane Labeling (Sigma-Aldrich) following the manufacturer’s protocol. Briefly, EVs were resuspended in Diluent C following final centrifugation. EVs were labeled with the PKH67 dye, incubated for 5 min and the staining was stopped with 1% BSA in PBS. Labeled EVs were washed with PBS prior to addition to MCF-7 for overnight (>14 h) incubation. Proliferating cells were identified using a Ki-67 (sc-23900) (Santa Cruz Biotechnology, Inc., Dallas, TX, USA) and Alexa Fluor 555 goat anti-mouse IgG (H + L) (A21422) (Invitrogen, Waltham, MA, USA) antibodies according to the manufacturer’s protocol. Cells were stained with DAPI for 10 min prior to imaging on a 20× objective using a Nikon C2+ confocal microscope (Nikon Instruments, Melville, NY, USA) in The University of Alabama Optical Analysis Facility.

### 2.9. Flow Cytometry

Flow cytometry was performed using a Bio-Rad S3e Cell Sorter (Bio-Rad Laboratories, Hercules, CA, USA). CSC marker expression was tested using PE Mouse Anti-Human CD24 Clone ML5 (RUO), FITC Mouse Anti-Human CD44 Clone G44-26 (also known as C26) (RUO), PE Mouse IgG2a, κ Isotype Control Clone G155-178 (RUO), FITC Mouse IgG2b κ Isotype Control Clone 27-35 (RUO) (BD Biosciences, Franklin Lakes, NJ, USA, cat.# 555428, 555478, 555574, and 555742, respectively) antibodies according to the manufacturer’s protocol. Proliferating cells were measured using a Ki-67 (sc-23900) (Santa Cruz Biotechnology, Inc., Dallas, TX, USA) and Alexa Fluor 555 goat anti-mouse IgG (H + L) (A21422) (Invitrogen, Waltham, MA, USA) antibodies according to the manufacturer’s protocol.

### 2.10. Measurement of ATP Production

Cellular ATP production was measured using the ATPlite™ Luminescence Assay System (PerkinElmer, Waltham, MA, USA) as previously described [43,44]. Briefly, cells (0.1 × 10^6^/well) were lysed on the shaker for 5 min. Cells were incubated with the substrate (luciferin) on the shaker for 10 min. The reaction between ATP, luciferase and luciferin produced bioluminescence. ATP-induced luminescence was measured using a fluorescence microplate reader (CLARIOstar, BMG Labtech, Mornington, Australia).

### 2.11. Statistical Analyses

Results were analyzed using the Student’s unpaired *t*-test and one-way ANOVA (GraphPad Prism, La Jolla, CA, USA). Tukey and Fisher tests were used to determine significance, with *p*-values < 0.05 presenting as significant.

## 3. Results

### 3.1. Direct Flow Filtration Produces Higher EV Recovery Than Differential Ultracentrifugation

**Figure 1:**
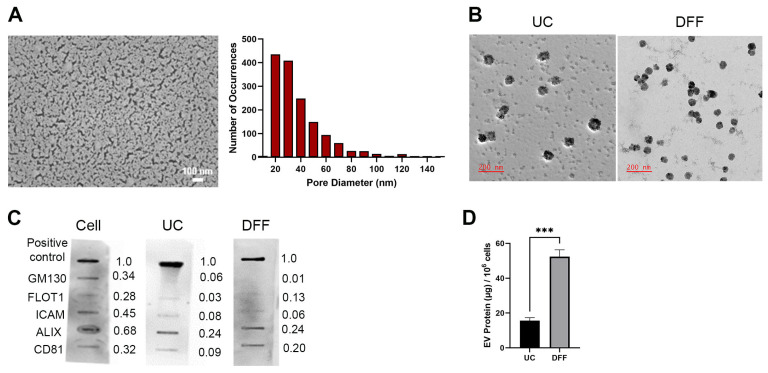
Verification of isolation of extracellular vesicles (EVs) produced by serum-free MDA-MB-231 cells. (**A**) Representative top surface scanning electron micrograph (FE-SEM) of a pre-filtration Synder LY membrane used for EV isolation (left; scale bar: 100 nm) and histogram demonstrating size distribution of pore sizes (right). (**B**) Representative transmission electron micrograph (TEM) image of EVs derived using ultracentrifugation (UC; left) and of EVs derived using direct flow filtration (DFF; right). (**C**) Exosome Antibody Array of MDA-MB-231 whole cell lysate, UC-derived EV proteins, and DFF-derived EV proteins (50 µg each). Numbers represent relative intensity of the bands compared to the positive control. Original Western blot images are available in Supplementary Materials. (**D**) Total protein isolated from EVs per one million cells. *n* ≥ 3; mean ± standard error; *** *p* < 0.001 via Student’s *t*-test with Welch corrections.

**Figure 2:**
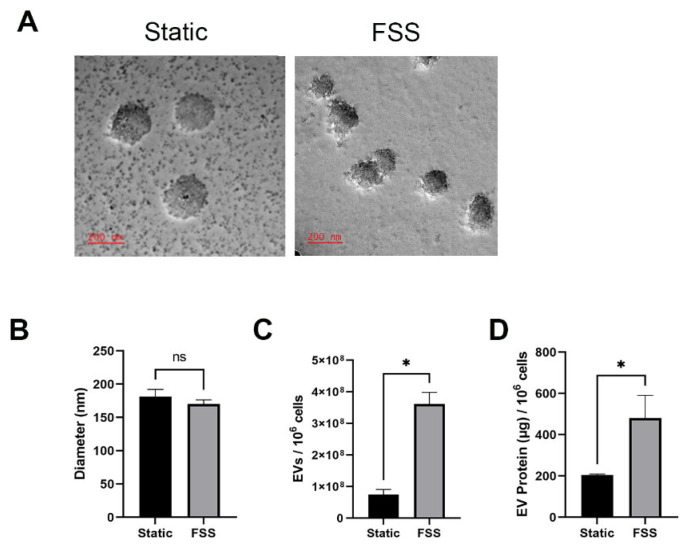
Impact of FSS on EVs from serum-free MDA-MB-231 cells. (**A**) TEM images of EVs derived from static and FSS cultures. Scale bars: 200 nm. (**B**) Mean particle size of EVs based on NTA. (**C**) Concentration of EVs derived from static and FSS cultures determined through NTA. (**D**) EV protein isolated following ethanol precipitation. *n* ≥ 3; mean ± standard error; * *p* < 0.05; ns = not significant via Student’s *t*-test with Welch corrections.

To compare a traditional EV-isolation method with our novel approach, we collected EVs produced in MDA-MB-231 serum-free media by differential ultracentrifugation (UC) and direct flow filtration (DFF; Figure 1A). The results of the top surface image analysis indicate that almost all of the pores are <100 nm. This confirmed that the membrane PES pore sizes are smaller than those of EVs (>150 nm and <200 nm, Figure 2), allowing the EVs to be collected and concentrated as the retentate. We characterized the EVs from both isolation methods by TEM and protein analysis. The size and morphology of the EVs derived was consistent across both methods (Figure 1B). To further support successful isolation of EVs, we performed protein analysis to assess the absence of contaminating cellular proteins and the presence of established exosome markers. Both the UC-and DFF-derived EVs were negative for the cell marker GM130 and showed comparable amounts of the cytosolic exosome marker ALIX, but the DFF EVs demonstrated a relatively higher concentration of CD81 (Figure 1C). For more substantive evidence of increased EV recovery, we measured the total protein isolated from both methods (Figure 1D). DFF EVs produced a higher mass of total protein, suggesting an overall higher recovery. This is supportive of using DFF as a novel method of EV isolation since the qualities of the isolated particles are consistent with the traditional centrifugation method, but with the advantages of a higher recovery and a significantly less laborious, varied, and time-consuming process.

### 3.2. Fluid Shear Stress Increases the Quantity of EVs Produced

Cellular stress is known to affect the production and composition of EVs, so we sought to investigate the impact of FSS on the size and quantity of EVs produced by serum-free MDA-MB-231 cells. Although the sizes of EVs did not change (Figure 2A,B), nanoparticle tracking analysis revealed that cells under FSS produced nearly triple the amount of EVs than the static control (Figure 2C). The results were further supported by the total protein quantification of EVs derived from static and FSS cultures (Figure 2D). Together, those data suggest that FSS induced increased the production of EVs.

### 3.3. Fluid Shear Stress Increases Stemness-Related miRNA in EVs

**Figure 3:**
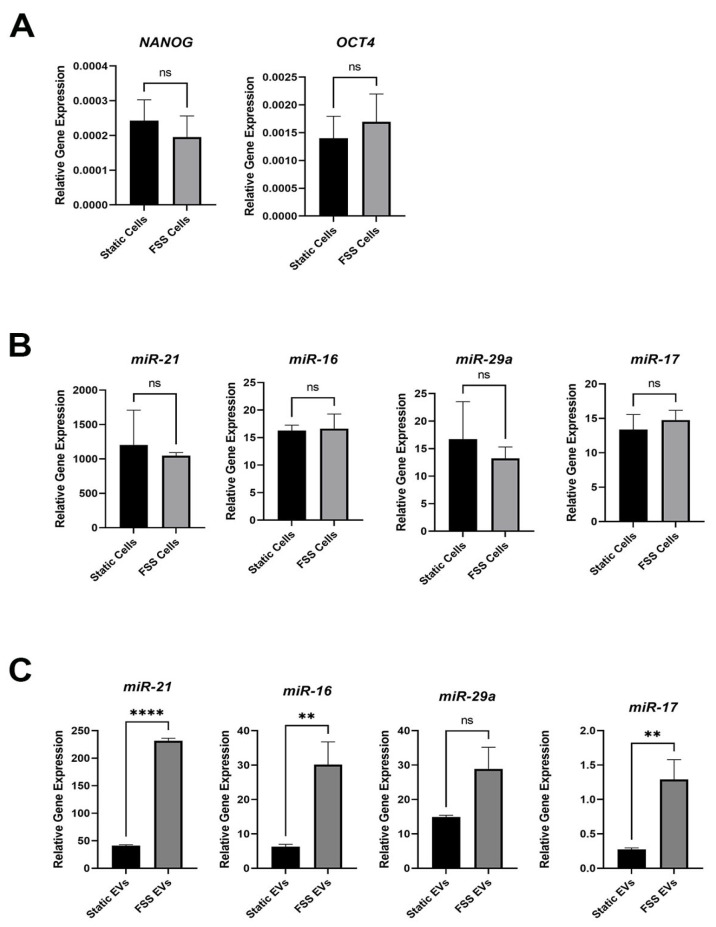
Expression of stemness markers and microRNAs in serum-free MDA-MB-231 cells and corresponding EVs with and without FSS. (**A**) RNA levels of stemness-related genes *NANOG* and *OCT4* normalized to *ACTB*. (**B**) miRNA levels in static- and FSS-cultured MDA-MB-231 cells and (**C**) in their corresponding EVs normalized to reference miRNA marker miR-30e. *n* = 3 biological replicates each with 2 or 3 technical replicates; mean ± standard error; ** *p* < 0.01; **** *p* < 0.0001; ns = not significant via Student’s *t*-test.

Previous work showed that the application of FSS can enrich the CSC-like subpopulation (CD44^+^/CD24^−^) in MCF-7 breast cancer cells but not in MDA-MB-231 breast cancer cells that already have a high percentage of CSC-like subpopulation [45]. Here, we observed a similar phenotype, where FSS did not increase the expression of stemness markers *NANOG* and *OCT4* in MDA-MB-231 (Figure 3A). The same was true for various miRNAs (miR-21, miR-17, miR-16, and miR-29a) that are known to mediate stemness and/or metastasis (Figure 3B). Interestingly, when analyzing for the same stemness-related miRNAs in the isolated EVs, there was a statistically significant increased expression of them (except for miR-29a) in those from the shear stressed cells (Figure 3C). These results suggest that FSS EVs have increased miRNA cargos that can modulate stemness to their neighboring cells.

### 3.4. MCF-7 Cells Receiving EVs from MDA-MB-231 Showed Increased Stem-Like Signature

**Figure 4:**
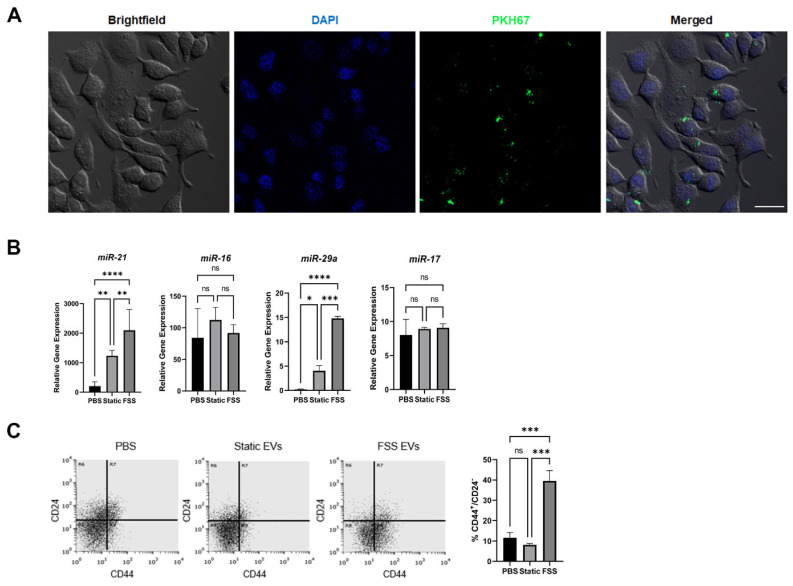
Impact of EVs derived from CSC-like MDA-MB-231 cells on MCF-7 cells. (**A**) Uptake of PKH67-labeled EVs in MCF-7 cells. Left to right: brightfield, DAPI nuclear stain, PKH67-labeled EVs, and merged. Scale bar: 25 µm. (**B**) miRNA expressions of stemness-related markers in MCF-7 cells after 24 h incubation with PBS control (PBS), static cell-derived EVs (static), and FSS-derived EVs (FSS). Expressions were normalized to reference miRNA marker miR-30e. *n* = 3 biological replicates each with 2 or 3 technical replicates; mean ± standard error; * *p* < 0.05; ** *p* < 0.01; *** *p* < 0.001; **** *p* < 0.0001; ns = not significant via one-way ANOVA. (**C**) Cancer stem cell-like subpopulation (CD44^+^/CD24^−^; lower right quadrant) of MCF-7 cells after 24 h of EV introduction measured via flow cytometry (*n* = 3).

We hypothesized that the uptake of EVs from MDA-MB-231 would transform the recipient cells, particularly towards an increased stem-like phenotype. To test this, EVs collected from static and FSS-treated MDA-MB-231 were labeled with fluorescent membrane label PKH67 and incubated overnight with unlabeled MCF-7 cells in a serum-depleted culture medium. We observed the successful uptake of these PKH67-labeled EVs in MCF-7 recipient cells after 24 h (Figure 4A). In particular, we observed EV agglomeration on the same *Z*-plane as the cell nucleus contrary to a halo around the nucleus, suggesting cellular uptake of EVs and their retention within the cells. We then analyzed the miRNA in the MCF-7 cells after incubation with the EVs. Significantly, both miR-21 and miR-29a showed increased expression in the recipient cells, especially for those with FSS EVs (Figure 4B). We further assayed for the CSC-like CD44^+^/CD24^−^ population. Interestingly, this subpopulation was only significantly increased in FSS EV-treated MCF-7 cells (Figure 4C), suggesting that not all stemness-related miRNA changes directly correlate to changes in CD44^+^/CD24^−^ subpopulation. Nevertheless, these results suggest that the EV uptake does indeed transform the recipient cells into an increased stem-like signature.

### 3.5. MCF-7 Cells Receiving EVs from MDA-MB-231 Showed Increased Proliferation and ATP Production

**Figure 5:**
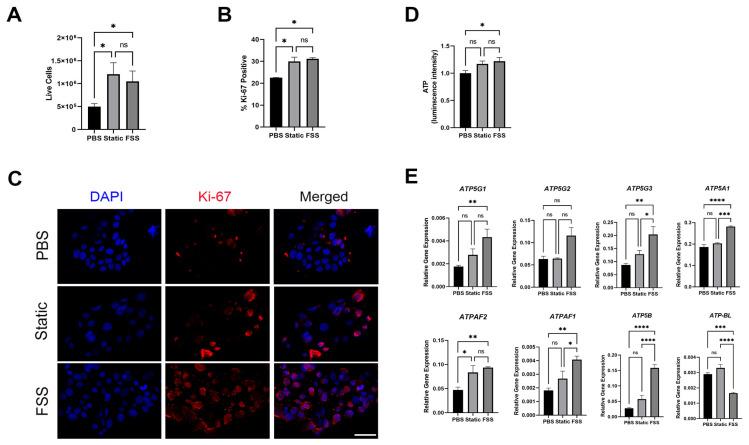
Impact on MCF-7 proliferation 24 h post-EV introduction. (**A**) Live cell counts of MCF-7 with PBS control (PBS), static cell-derived EVs (static), and FSS-derived EVs (FSS). (**B**) Percent-positive Ki-67 populations quantified using flow cytometry. (**C**) Ki-67 staining indicating proliferating cells. Left to right: DAPI nuclear stain, Ki-67, and merged. Scale bar: 50 µm. (**D**) ATP measurements in MCF-7 cells quantified using luminescence. (**E**) RNA levels of F_1_Fo ATP synthase c-subunit genes (*ATP5G1*, *ATP5G2*, and *ATP5G3*), α-subunit genes (*ATP5A* and *ATPAF2*), and β-subunit genes (*ATPAF1*, *ATP5B*, and *ATP-BL*) normalized to housekeeping gene *ACTB*. *n* = 3 biological replicates each with 2 or 3 technical replicates; mean ± standard error; * *p* < 0.05; ** *p* < 0.01; *** *p* < 0.001; **** *p* < 0.0001; ns = not significant via one-way ANOVA.

Upon collecting the MCF-7 cells, it was noted that the samples treated with the EVs showed a higher number of live cells than the negative control (Figure 5A). Indeed, there was a higher percentage of Ki-67-postive proliferative cells when treated with either static EVs or FSS EV cells compared to the untreated control (Figure 5B,C). To test whether the EV-induced changes in proliferation were associated with energy metabolism, we measured the amounts of ATP produced in the MCF-7 cells (Figure 5D). The samples treated with the FSS EV showed a significant increase in intracellular ATP levels compared to the untreated control. This was further corroborated when assaying for the expressions of genes encoding for the α-, β-, and c-subunits of F_1_Fo ATP synthase (Figure 5E), the enzyme complex that governs cellular energy metabolism. Overall, the results showed a statistically significant increase in the α- and c-subunit genes in the FSS EV-treated MCF-7 cells compared to the untreated control.

## 4. Discussion

In this study, we compared traditional EV isolation method via UC to a novel approach via DFF. Furthermore, we explored the impact that FSS has on EV production and on the miRNAs contained within the EVs, as well as the translated effects of those EVs on recipient MCF-7 cells.

We observed that DFF was superior to traditional differential UC in recovering higher quantity EVs without impacting their quality (Figure 1). UC is a laborious and highly variable method in isolating EVs [46]. In contrast, DFF allows for large-scale isolation of EVs with higher purity [47,48], motivating our approach. Presently, there is a high interest to adopt EVs as targeted therapeutic delivery vehicles, so it is advantageous to identify and validate a scalable and reproducible EV isolation process. With TEM, we showed that the EVs isolated from both methods produced comparable particles of similar size and morphology, consistent with previously reported imaging data [49]. Interestingly, protein marker analysis indicated EVs had similar levels of ALIX, but higher levels of CD81 via DFF than via UC. It is possible that DFF preferentially selects for CD81-positive EVs, and this will need to be explored further in future studies.

We hypothesized that FSS would increase the production of EVs like other previously reported stresses [26]. While the bulk population size of the EVs did not significantly change (Figure 2A,B), there was a significant increased production of EVs due to the FSS (Figure 2C). The static culture cells produced approximately 100 EVs per cell, whereas the FSS-exposed cells produced 350 EVs per cell. Thus, our results suggest that sublethal FSS can have beneficial effects in the biomanufacturing of EVs.

We previously demonstrated that the application of FSS preferentially enriched the CSC-like CD44^+^/CD24^−^ subpopulation in MCF-7 but not in the more aggressive MDA-MB-231 [45,50]. Here, key stemness-related genes and miRNAs did not change within the cells after FSS, but the miRNA cargos in the secreted EVs showed a significant increase (Figure 3). To our knowledge, this is the first reporting of such an effect by FSS on EVs derived from breast cancer cells. Previous studies showed that hypoxic and serum deprivation stresses impact the composition of EVs [23,24,25,26]. Consistent with the previous literature that demonstrated upregulation of miR-21 and miR-29a in breast cancer stem cells [28], our results suggest that EVs are one of the key means by which such changes occur after FSS. Together, these results suggest that FSS can improve the survivability of the cells via EVs [51], while also communicating with the nearby cells through them.

It is particularly interesting that miR-21 showed such a high increase upon EV uptake (Figure 4A,B), as it is correlated to numerous oncological processes including invasion, migration, and targeting of tumor suppressor genes associated with proliferation [52,53]. miR-21 has previously been shown to localize in mitochondria, where it upregulates cytochrome B, ultimately resulting in increased ATP production [54]. miR-21 also has multiple genetic targets including tumor suppressors *PTEN* and *PCDC4*, which play critical roles in CSC maintenance [55,56,57]. Additionally, miR-21 has been shown to play a role in the regulation of cellular energy metabolism in cancer-associated fibroblasts [58,59]. Our data suggest that increased miR-21 in receiving cells is indeed correlated to increased stem-like signature (Figure 4C), proliferation, and energy metabolism (Figure 5), suggesting a potential role of miR-21 favoring cancer cell energy metabolism. We further postulate that the transfer of miR-21, using EVs as a key delivery vehicle and mechanism of communication, is an important mechanism by which metastasizing cancer cells potentially ward off chemotherapies and pre-condition their microenvironment to increase stem cell-like characteristics.

We have previously reported that MCF-7 cells treated with FSS, without EVs, caused the metabolic remodeling of cancer cells by decreasing the c- and β-subunit of the F_1_Fo ATP synthase [44]. The c-subunit is responsible for forming a leak channel [58]; thus, FSS-mediated c-subunit reduction may improve metabolic efficiency. Interestingly, recent studies suggested that the leak channel can be regulated by the conformational changes in the F_1_Fo ATP synthase [60]. Notably, dissociation of the c-subunit from the F_1_ complex is shown to be associated with a large channel conductance. Therefore, it is possible that the upregulation of the F_1_ complex components such as the α- and β-subunits may prevent the opening of the leak channel by supporting the assembly of the F_1_Fo ATP synthase. Future studies investigating the ratio of the F_1_Fo ATP synthase subunits may help elucidate the underlying mechanisms of altered energy metabolism in EV-exposed cancer cells.

## 5. Conclusions

In conclusion, we have demonstrated that DFF is an effective method for isolating EVs at a higher yield than traditional UC. We have also shown that the application of FSS impacts the stemness-related miRNA cargos in EVs, namely miR-21. Furthermore, the incubation of these EVs from stem cell-like and aggressive breast cancer cells can result in an increased expression of miR-21 and miR-29a in the recipient cells to ultimately increase the CSC-like population and proliferative cells. Thus, our studies highlight the importance of physiological FSS in EV production and the critical role EV-mediated transfer of key microRNAs may play in the “seed-and-soil” mechanisms of cancer metastasis.

## Figures and Tables

**Table 1:** Primer sequences used in this study.

Primer	Sequence
*ACTB* forward	GCC CTG GAC TTC GAG CAA GAG A
*ACTB* reverse	ATG GTG ATG GAC CTG GCC GTC A
*ATP5A1* forward	TGG AGC CCA GCA AGA TTA CA
*ATP5A1* reverse	TGA TAG TGC CCA ACA AGG CT
*ATP5B* forward	CGC AAA CAT CTC CTT CGC CA
*ATP5B* reverse	AGT CCC TCA TCA AAC TGG ACG
*ATP5G1* forward	GTG AGT CTG TCA CCT TGA GCC
*ATP5G1* reverse	CTG CAC TCC TAC TAC CCT GCA A
*ATP5G2* forward	GTC AAG AGC ACC TCA CAG C
*ATP5G2* reverse	TCT GTC AGT ATC TCC GGT CGT
*ATP5G3* forward	TTA ATG GGG CCC AGA ATG GTG
*ATP5G3* reverse	CCA GCC ACT CCT ACT GTT GC
*ATPAF1* forward	TAT GTG CTC TGC CAA GAA GGG
*ATPAF1* reverse	AAG TGG AGT TCA GTA CCT GTC C
*ATPAF2* forward	TTG AAG AAA CTG GGC GTG TC
*ATPAF2* reverse	CTG CTT GAA CAT TCC TCA GCC
*ATP*-*BL* forward	ACC TCA CAT CTG ACC CTG GA
*ATP*-*BL* reverse	AAG CTT CCC TTC TTG GCC TC
*NANOG* forward	AAT ACC TCA GCC TCC AGC AGA TG
*NANOG* reverse	TGC GTC ACA CCA TTG CTA TTC TTC
*OCT4* forward	GAG AAC CGA GTG AGA GGC AAC C
*OCT4* reverse	CAT AGT CGC TGC TTG ATC GCT TG

## Data Availability

The original contributions presented in the study are included in the article/Supplementary Materials, and further inquiries can be directed to the corresponding author. The raw data supporting the conclusions of this article will be made available by the authors on request.

## Supplementary Materials

The following supporting information can be downloaded at: https://www.mdpi.com/article/10.3390/biom14070757/s1, Original Western blot images.
