# Supplementary material for: Extracellular Vesicle-Mediated Modulation of Stem-like Phenotype in Breast Cancer Cells under Fluid Shear Stress
Source: Biomolecules. 2024 Jun 25;14(7):757. doi: 10.3390/biom14070757 (PMC11274421; doi:10.3390/biom14070757)

Exo-Check™ Exosome Antibody Array – Full Blots

MDA-MB-231 Cell Lysate

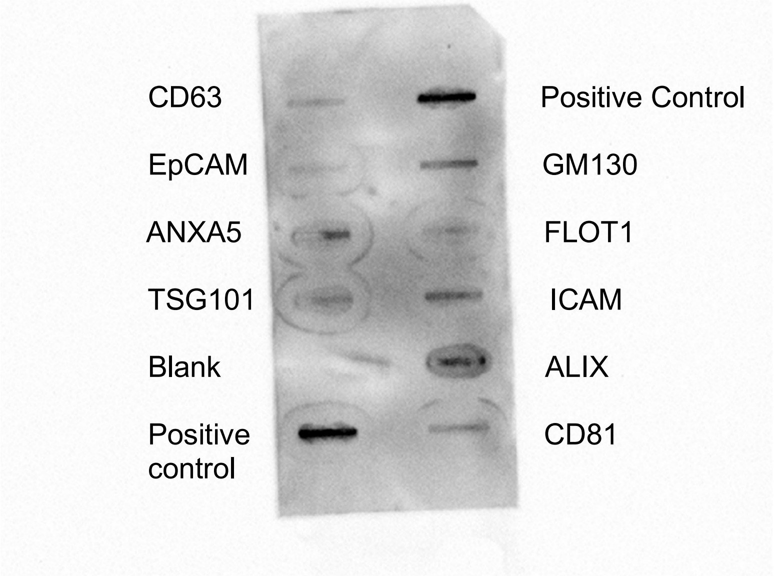

MDA-MB-231 Extracellular Vesicles Ultracentrifuge

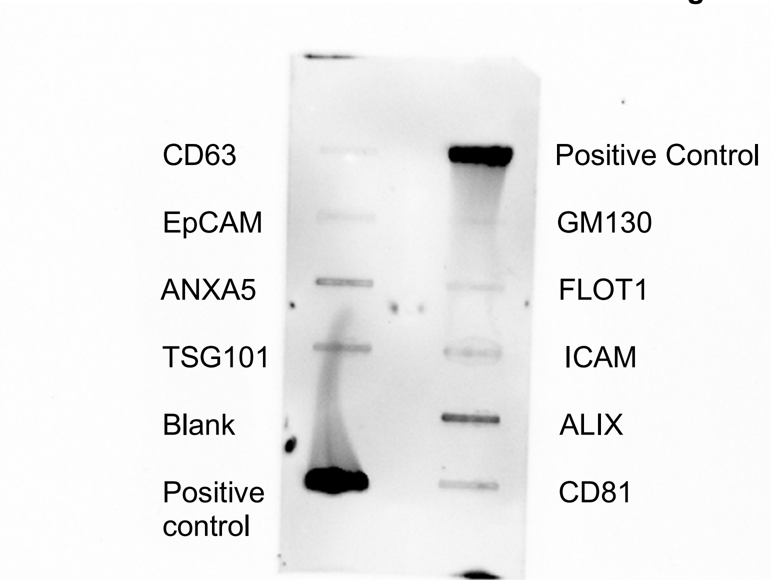

MDA-MB-231 Extracellular Vesicles Direct Flow Filtration

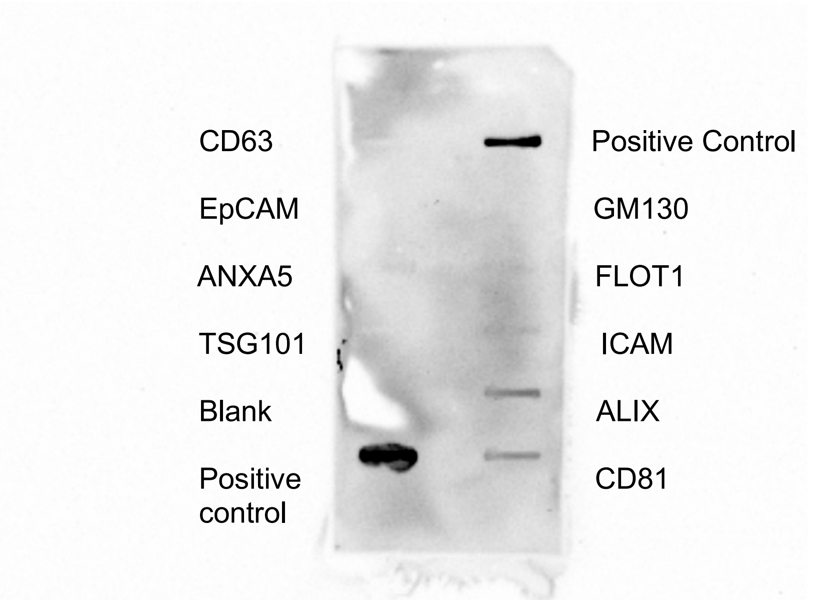

Supplement: Supplementary file 1 [file biomolecules-14-00757-s001.zip › biomolecules-3043456-supplementary.pdf]
